# Mechanistic insights from experimental autoimmune encephalomyelitis into immune regulation, autophagy, gut microbiota, blood-brain barrier integrity, and NLRP3 inflammasome-mediated pyroptosis in multiple sclerosis: potential clinical implications

**DOI:** 10.3389/fnins.2026.1803309

**Published:** 2026-04-29

**Authors:** Shu-Min Zhang, Wen-Chao Tang, Fang-Fei Liu, Qian-Qian Bai, Yan Guo, Xiao-Zhi Bai, Ying-Fei Wang, Xiao-Yu Ma, Shun-Ji Pan, Yan-Hui Yang, Gan-Qin Du, Hua Fan

**Affiliations:** 1 The First Affiliated Hospital, and College of Clinical Medicine of Henan University of Science and Technology, Luoyang, China; 2 Department of Emergency Medicine, The First Affiliated Hospital, and College of Clinical Medicine of Henan University of Science and Technology, Luoyang, China; 3 Department of Neurology, The First Affiliated Hospital, and College of Clinical Medicine of Henan University of Science and Technology, Luoyang, China; 4 Luoyang Key Laboratory of Neuroimmunology and Innovative Drug Screening, Luoyang, China; 5 Office of Research & Innovation, The First Affiliated Hospital, and College of Clinical Medicine of Henan University of Science and Technology, Luoyang, China

**Keywords:** autophagy, blood-brain barrier, drugs, experimental autoimmune encephalomyelitis (EAE), gut microbiota, immunomodulation, multiple sclerosis (MS), NLRP3 inflammasome

## Abstract

Multiple sclerosis (MS) is a chronic autoimmune disorder characterized by inflammatory demyelination in the central nervous system, predominantly presenting in young adults, with a steadily increasing global incidence. In China, MS is classified as a rare disease and imposes a considerable medical and socioeconomic burden. Current clinical management mainly relies on immunomodulatory and immunosuppressive therapies; however, limitations in long-term efficacy, safety, and economic cost highlight the need for a more comprehensive understanding of disease mechanisms. Experimental autoimmune encephalomyelitis (EAE) is a widely used animal model for investigating the immunopathological basis of MS. Although EAE does not fully replicate the heterogeneity and long-term progression of human MS, it provides an important experimental framework for elucidating specific molecular and cellular pathways involved in disease development. This review synthesizes mechanistic evidence derived from EAE studies, focusing on immune regulation, autophagy modulation, gut microbiota-brain axis interactions, maintenance of blood-brain barrier integrity, and inhibition of NLRP3 inflammasome-mediated pyroptosis. By integrating findings within these defined pathological domains, this work aims to clarify how modulation of these interconnected pathways contributes to the present understanding of MS pathogenesis and to discuss their potential clinical relevance. These findings not only enhance our understanding of MS pathogenesis but also provide a foundation for developing multi-target, synergistic therapeutic strategies.

## 1 Introduction

Multiple sclerosis (MS) is an autoimmune disorder that affects the central nervous system (CNS) (Calahorra et al., 2022), characterized by focal demyelination in the white matter (Adamczyk and Adamczyk-Sowa, 2016), inflammatory cell infiltration (Adamczyk-Sowa et al., 2016), and often accompanied by axonal injury, oligodendrocyte apoptosis, and heightened oxidative stress (Avila et al., 2023; Brambilla, 2019; Fazeli et al., 2023). Globally, over 2.8 million people are affected by MS, with peak onset occurring during the ages of 29–39, and a significantly higher incidence in females compared to males (male-to-female ratio approximately 1:1.5–1:2). Recent years have seen an upward trend in MS incidence. In China, the national incidence rate among hospitalized patients is 0.235 per 100,000 person-years, with a male-to-female ratio of 1:2.02. In light of its severity, MS was included in China's “First Batch of Rare Disease Catalog” in 2018. Clinically, MS often presents with a range of neurological deficits, such as limb paralysis, ataxia, sensory disturbances, and urinary/fecal incontinence (Pinke et al., 2020). Some of which can be reversible in the early stages of the disease. However, as the disease progresses, irreversible disability may develop. Fatigue, a common and debilitating symptom, also significantly impacts the quality of life in MS patients. MS has no cure, severely affecting patients' quality of life and imposing significant socioeconomic burdens (Sand, 2015; Michaličková et al., 2019; Thompson et al., 2018; van den Hoogen et al., 2017).

Current global prevention and treatment strategies for MS remain suboptimal (Zhong et al., 2017; Witte et al., 2014), as its precise etiology and pathogenesis have yet to be fully understood. Despite advances in diagnostic and therapeutic strategies, current treatments for MS remain limited in their ability to fully prevent disease progression or promote durable neurorepair. These limitations underscore the need to further explore therapeutic approaches with improved target specificity and well-characterized safety profiles. In this context, investigating diverse bioactive molecules may provide additional mechanistic insights; however, their potential advantages in terms of safety and efficacy should be interpreted cautiously and require preclinical and clinical validation.

In MS research, commonly used animal models include experimental autoimmune encephalomyelitis (EAE), toxin-induced demyelination models, and virus-induced demyelination models. Among these, the EAE model is considered the most ideal due to its close resemblance to MS in biochemical characteristics, immune responses, pathological changes, and clinical manifestations. As such, it serves as a valuable model for investigating MS pathogenesis and evaluating potential therapeutic strategies. This review, focused on the EAE model, systematically explores the molecular mechanisms and intervention strategies in MS, offering significant research insights.

In recent years, advancements in immunology and neuroscience have deepened our understanding of MS pathogenesis and therapeutic approaches. In addition to the classical mechanisms of immune-inflammatory responses and blood-brain barrier (BBB) disruption, emerging pathological processes such as autophagy imbalance, gut microbiota dysbiosis, and NLRP3 inflammasome hyperactivation have been shown to contribute to disease progression in EAE model studies. For example, autophagy dysfunction exacerbates neuroinflammation, while gut microbiota influences the CNS via the “gut-brain axis”, promoting inflammation and neurodegeneration. NLRP3 inflammasome activation and subsequent pyroptosis further intensify neurological damage.

**Figure 1:**
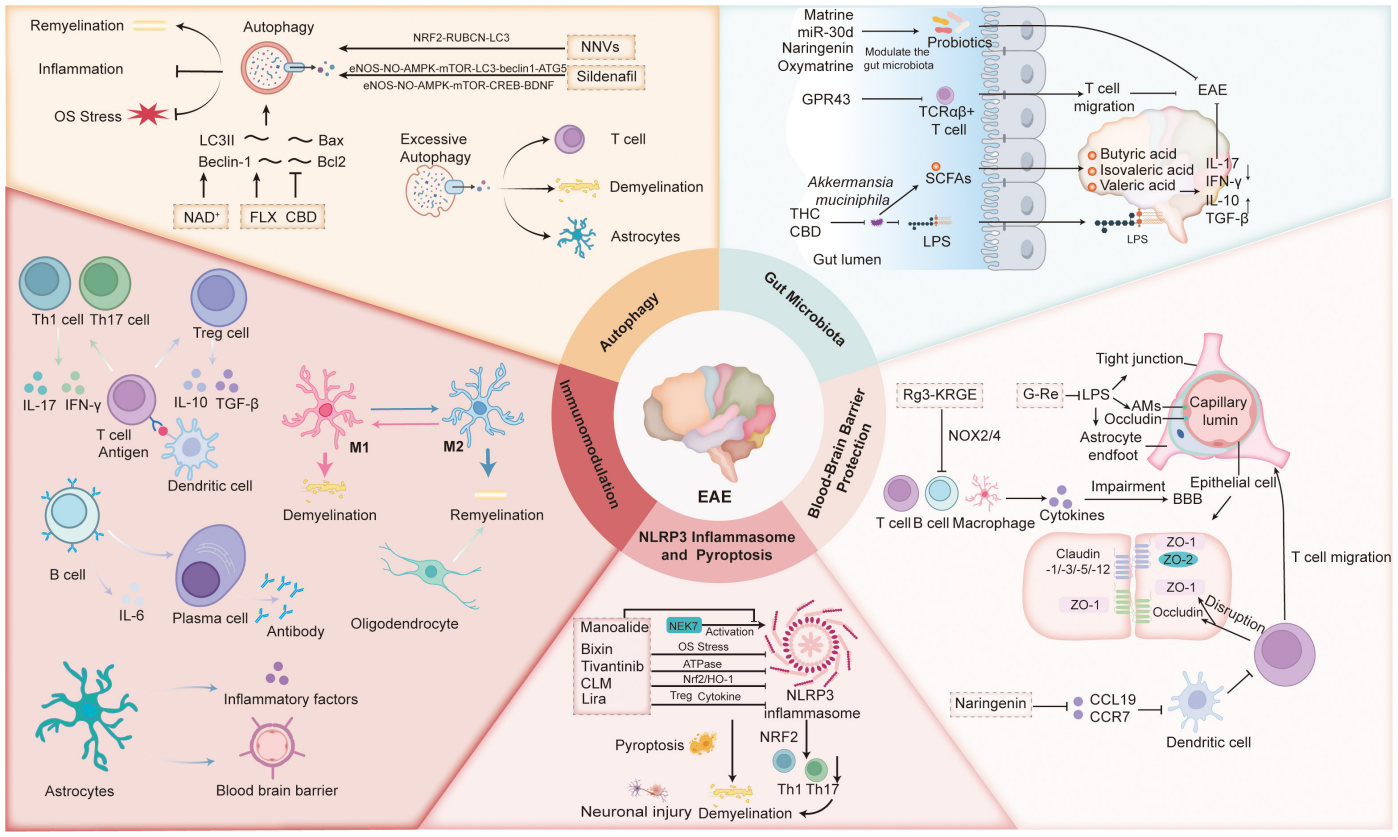
Multiple potential compounds target key pathological processes in multiple sclerosis (MS) through mechanisms including immune modulation, autophagy regulation, gut microbiota and microenvironment intervention, blood-brain barrier (BBB) enhancement, and inhibition of NLRP3 inflammasome activation and pyroptosis, thereby delaying disease progression and improving clinical symptoms in experimental autoimmune encephalomyelitis (EAE). Additional abbreviations: M1, M1-type macrophage; M2, M2-type macrophage; Th1, T helper type 1; Th17, T helper type 17; Treg, regulatory T cell; IL-10, interleukin 10; IL-17, interleukin 17; IL-6, interleukin 6; TGF-β, transforming growth factor beta; IFN-γ, interferon gamma; LC3II, microtubule-associated protein 1 light chain 3 II; FLX, fluoxetine; CBD, cannabidiol; NRF2, nuclear factor erythroid 2-related factor 2; RUBCN, rubicon; Bcl2, B-cell lymphoma 2; Bax, Bcl-2-associated X protein; eNOS, endothelial nitric oxide synthase; NO, nitric oxide; AMPK, AMP-activated protein kinase; mTOR, mammalian target of rapamycin; CREB, cAMP response element-binding protein; BDNF, brain-derived neurotrophic factor; NLRP3, NLR family pyrin domain containing 3; NEK7, NIMA-related kinase 7; HO-1, heme oxygenase 1; LPS, lipopolysaccharide; SCFAs, short-chain fatty acids; EAE, experimental autoimmune encephalomyelitis; TCRαβ+, T cell receptor alpha beta positive; NAD+, nicotinamide adenine dinucleotide; miR-30d, microRNA 30d; THC, tetrahydrocannabinol; GPR43, G protein-coupled receptor 43; NOX2/4, NADPH oxidase 2/4; CCL19, C-C motif chemokine ligand 19; CCR7, C-C chemokine receptor type 7; ZO-1, zonula occludens-1; ZO-2, zonula occludens-2; BBB, blood-brain barrier.

Building on these findings, this review systematically summarizes recent advances in preclinical MS drug research over the past five years, focusing on the mechanisms through which various compounds intervene in critical MS pathological processes. These mechanisms include immune modulation (targeting T cells, microglia/macrophages, dendritic cells, B cells, etc.), autophagy regulation, gut microbiota and microenvironment modulation, BBB enhancement, and inhibition of NLRP3 inflammasome activation and pyroptosis (Figure 1). These strategies alleviate disease progression and improve clinical outcomes, providing a theoretical foundation for future MS drug development and advancing this field toward better patient prognosis and quality of life.

## 2 Challenges in MS treatments

Therapeutic strategies for MS have evolved significantly, transitioning from initial acute-phase symptomatic management to current long-term management protocols focused on Disease-Modifying Therapy (DMT). Prior to the 1990s, clinical treatment mainly relied on short-term, high-dose glucocorticoid pulse therapy during acute episodes. The introduction of injectable therapies, such as interferon-β and glatiramer acetate, established the early standard for managing remission-phase Relapsing-Remitting MS (RRMS).

With the advent of oral DMT medications and monoclonal antibody drugs in the 21st century, MS treatment objectives shifted fundamentally. The focus moved from managing acute-phase symptoms to achieving long-term disease modification, primarily by reducing relapse rates and delaying disability progression. This transformation also led to updates in diagnostic and treatment protocols. For example, the “2023 Edition of Guidelines for Diagnosis and Treatment of MS” recommends initiating DMT as early as possible upon diagnosis and maintaining long-term treatment, with individualized protocols based on disease severity, drug safety profiles, efficacy, economic factors, and patient preferences. Treatment efficacy is assessed by the goal of “no evidence of disease activity”, using core indicators such as annualized relapse rate, Expanded Disability Status Scale (EDSS) scores, MRI lesion activity, and brain volume changes.

MS clinical treatment is currently divided into two phases: treatment of acute relapse and long-term disease-modifying treatment.Relapse treatment aims to alleviate symptoms and improve neurological deficits, primarily using glucocorticoids, occasionally also plasma exchange, and intravenous immunoglobulin. DMT, focuses on suppressing disease activity and delaying overall disease progression. First of all, DMTs inhibit disease activity, but we still have a problem with slowing down progression. The U.S FDA has approved multiple DMTs for multiple sclerosis. Current clinical practice categorizes these agents by efficacy, broadly distinguishing moderate- and high-efficacy therapies. Moderate-efficacy treatments include interferon β formulations, glatiramer acetate, dimethyl fumarate, and teriflunomide. High-efficacy therapies comprise natalizumab, fingolimod, alemtuzumab, and most prominently, anti-CD20 monoclonal antibodies (e.g., ocrelizumab, ofatumumab, ublituximab), which have become central to contemporary treatment strategies. Mitoxantrone is now rarely used because of safety concerns.

However, existing therapeutic regimens exhibit notable limitations. Glucocorticoids, which are primarily used for short-term management of acute relapses, are associated with potential adverse effects such as infection, osteoporosis, and hepatic or renal dysfunction, particularly with repeated or high-dose exposure. While DMTs effectively modulate immune responses and have markedly improved disease control, their safety profiles vary across agents and generally remain acceptable with appropriate monitoring, including for infection and malignancy risks. In addition to safety concerns and cost considerations, treatment adherence and long-term tolerability are strongly influenced by factors such as route of administration, dosing frequency, and overall convenience. Currently approved therapies for MS, including immunomodulatory agents that interfere with peripheral immune cell activation, migration, or inflammatory cytokine release, primarily target peripheral immune responses and, to some extent, involve mechanisms related to immune regulation, inflammation suppression, and blood-brain barrier protection. However, these therapeutic approaches mainly aim to reduce relapse rates and delay disease progression, with limited direct effects on promoting remyelination, axonal protection, and tissue repair within the central nervous system, thus falling short of achieving effective neurological reconstruction or fundamental disease reversal.

In light of these limitations, research perspectives on MS have continued to expand. In recent years, studies on traditional Chinese medicine and natural bioactive compounds have suggested that, beyond modulating immune and inflammatory pathways already targeted by existing therapies, these agents may exert coordinated regulatory effects across multiple interconnected processes, including immune modulation, restoration of autophagic homeostasis, remodeling of the gut microbiota, maintenance of blood-brain barrier integrity, and inhibition of NLRP3 inflammasome activation. Through such network-based, multi-level regulation, they may influence both neuroinflammation and neurorepair processes. This review systematically summarizes preclinical evidence derived from EAE models, aiming to elucidate the novel mechanistic insights provided by these natural compounds in multi-target synergistic modulation, such as their roles in regulating immune-neural-gut axis interactions, coordinating autophagy and inflammasome crosstalk, and concurrently mediating anti-inflammatory and neuroprotective effects.

Furthermore, in evaluating the suppression of disease progression and repair-related outcomes, this review integrates multidimensional indicators reported in EAE studies, including improvements in clinical scores, reduction of demyelinated areas, decreased inflammatory cell infiltration, alterations in axonal injury markers, and restoration of myelin-related proteins such as MBP, in order to more objectively assess the potential value of traditional Chinese medicine and natural bioactive compounds in delaying disease progression and promoting neurorepair.

## 3 Mechanistic insights and therapeutic exploration in multiple sclerosis: lessons from the EAE model

### 3.1 Overview of animal models in ms and the central role of EAE

Given the limitations of current therapeutic strategies in promoting remyelination and achieving curative outcomes, animal models play a critical role in preclinical MS research, offering essential experimental platforms to explore effective treatment approaches. Currently, MS research primarily employs three types of animal models: EAE, toxin-induced demyelination, and virus-induced demyelination models (Bjelobaba et al., 2018; Procaccini et al., 2015; Smith, 2021; Dedoni et al., 2023). The EAE model is an immune-mediated MS model that effectively replicates key pathological features of MS, including CNS inflammation and demyelination, and reproduces clinical manifestations such as motor dysfunction and limb paralysis. This model is widely used to investigate MS pathogenesis and evaluate therapeutic strategies due to its strong representation of typical CNS inflammation, demyelination, and neurodegeneration (Dedoni et al., 2023). EAE induction methods include: (1) active immunization with myelin antigens (e.g., MOG, MBP) combined with complete Freund's adjuvant and pertussis toxin (Dedoni et al., 2023); (2) passive transfer induction, where encephalitogenic T cells from immunized donors are transferred to recipient animals (Dedoni et al., 2023); and (3) transgenic spontaneous induction, involving genetically modified mice expressing myelin antigen-specific T cell receptors, which develop the EAE phenotype spontaneously (Dedoni et al., 2023). Nevertheless, due to its high concordance with MS in clinical manifestations, pathological changes, and molecular characteristics, along with its excellent reproducibility and stability, the EAE model remains the internationally recognized standard for investigating MS pathogenesis and therapeutic strategies (Brambilla, 2019). Therefore, the EAE model holds irreplaceable value in elucidating MS immunopathological mechanisms and advancing therapeutic development.

### 3.2 Mechanistic insights and therapeutic exploration based on the EAE model

#### 3.2.1 Mechanistic evidence for immune modulation

**Figure 2:**
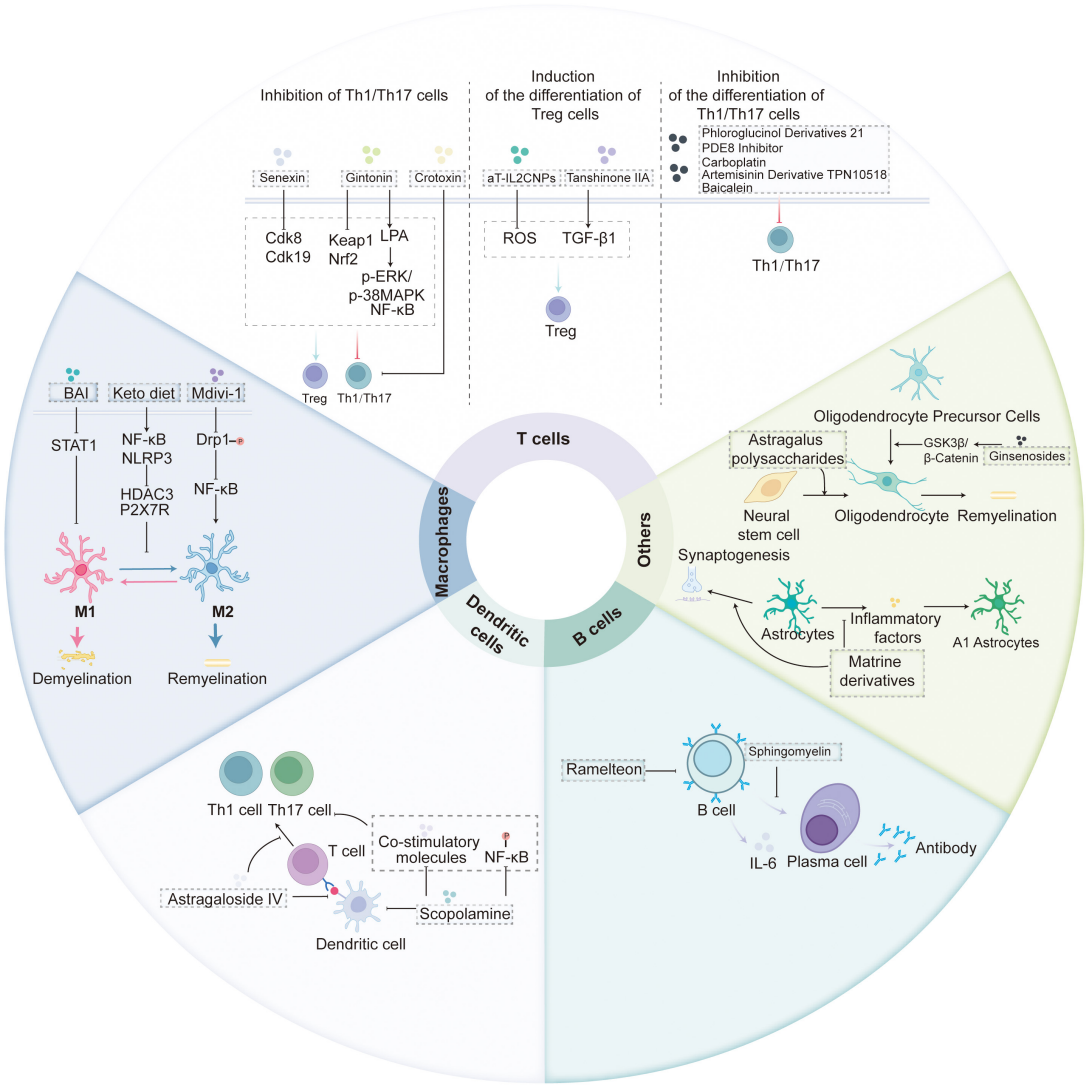
Multiple compounds exhibit neuroprotective effects through immunomodulatory actions of regulating T cell immune responses, inhibiting excessive microglial and macrophage activation, modulating dendritic cell antigen presentation, antagonizing B cell pathogenic effects, and influencing the functional states of oligodendrocytes and astrocytes. Additional abbreviations: NF- κ B, nuclear factor kappa-light-chain-enhancer of activated B cells; IL-6, interleukin 6; Th1, T helper type 1; Th17, T helper type 17; Treg, regulatory T cell; LPA, lysophosphatidic acid; Cdk8, cyclin-dependent kinase 8; Cdk19, cyclin-dependent kinase 19; aT-IL2CNPs, anti-T-cell interleukin-2-conjugated nanoparticles; Keap1, Kelch-like ECH-associated protein 1; Nrf2, nuclear factor erythroid 2-related factor 2; p-ERK, phosphorylated extracellular signal-regulated kinase; p-38MAPK, phosphorylated p38 mitogen-activated protein kinase; ROS, reactive oxygen species; TGF- β1, transforming growth factor beta 1; STAT1, signal transducer and activator of transcription 1; NLRP3, NLR family pyrin domain containing 3; Drp1, dynamin-related protein 1; HDAC3, histone deacetylase 3; P2X7R, P2X purinoceptor 7 receptor; M1, M1-type macrophage; M2, M2-type macrophage; BAI, brain angiogenesis inhibitor; Mdivi-1, mitochondrial division inhibitor 1; A1 Astrocytes, A1 reactive astrocytes; GSK3 β, glycogen synthase kinase 3 beta; PDE8, phosphodiesterase 8.

As a chronic inflammatory disease of the CNS, the pathogenesis of MS is closely linked to abnormal activation and interactions of various immune cells. Dysregulation of the immune system is considered the core pathogenic mechanism of MS, driving persistent inflammatory responses and secondary neuronal damage within the CNS. Recent studies have demonstrated that several compounds exhibit neuroprotective effects in EAE models through immunomodulatory actions. These compounds primarily act by regulating T cell immune responses, inhibiting excessive microglial and macrophage activation, modulating dendritic cell antigen presentation, antagonizing B cell pathogenic effects, and influencing the functional states of oligodendrocytes and astrocytes (Figure 2). These immunomodulatory strategies not only reduce inflammation and demyelination in EAE models but also provide valuable insights into the underlying mechanisms of MS pathology.

**Regulation of T cell-mediated immune responses** EAE is an autoimmune disease mediated by specifically sensitized CD4^+^ T cells, with typical pathological features including perivascular mononuclear cell infiltration in CNS microvessels and myelin loss. T cells play a central role in EAE development and progression by regulating immune responses and maintaining homeostasis through differentiation into various functional subsets (Shao et al., 2021), such as Th1, Th2, Th17, and regulatory T cells (Treg). Among these, Th1 and Th17 cells exacerbate inflammatory responses through the secretion of pro-inflammatory factors like IFN-γ and IL-17 (Racke et al., 2014), while Treg cells maintain immune balance by releasing anti-inflammatory factors, including IL-10 and TGF-β. An imbalance between these subsets is a key mechanism underlying abnormal autoimmune activation. Recent studies have identified several compounds that play pivotal roles in MS/EAE through the regulation of T cell differentiation, activation, and function. These mechanisms can be divided into three main categories. First, inhibition of Th1 and Th17 cell differentiation: compounds such as the phloroglucinol derivative Compound 21 (Zhao et al., 2020), phosphodiesterase-8 (PDE8) inhibitors (Basole et al., 2022), carboplatin (Lv et al., 2023), the artemisinin derivative TPN10518 (Xie et al., 2023), and baicalein (Ying et al., 2023) can inhibit the differentiation and function of Th1 and Th17 cells through various signaling pathways, thus reducing inflammatory responses and demyelination in EAE models. Second, promotion of Treg cell differentiation and function: ROS-scavenging nanovaccines and aT-IL2CNPs can induce antigen-specific Treg responses, while tanshinone IIA promotes Treg differentiation via the TGF-β1 pathway to delay EAE progression (Gong et al., 2020). Third, bidirectional regulation of Th1/Th17 and Treg balance: Gintonin modulates p-ERK/p-38 MAPK, NF-κB, and Keap1-Nrf2 signaling pathways through LPA receptor activation, simultaneously inhibiting CD4^+^ T cell proliferation, reducing Th1/Th17 numbers (Choi et al., 2021), and increasing Treg proportions. Crotoxin inhibits T cell proliferation and promotes Treg differentiation (Teixeira et al., 2020), while Cdk8/Cdk19 inhibitors such as CCT251921 and Senexin A also promote Treg-specific gene expression while inhibiting Th1/Th17 differentiation (Guo et al., 2019). These findings suggest that restoring the balance among Th1, Th17, and Treg cells may represent a relevant therapeutic direction in multiple sclerosis. In EAE models, these compounds modulate T cell subsets, attenuate inflammation, and reduce neuropathological damage, indicating a potential impact on disease progression. However, as these data are derived from preclinical studies, their clinical relevance and mechanisms require further validation.

**Regulation of microglial /macrophage immune responses** Microglia and macrophages, integral components of the mononuclear phagocyte system, play pivotal roles in immune regulation within the CNS. Their infiltration and polarization phenotypes are critically involved in the pathogenesis of MS and EAE. Studies have shown that microglia/macrophages can polarize into two main phenotypes: the classically activated M1 phenotype and the alternatively activated M2 phenotype. The balance between these phenotypes is a key regulatory point in MS/EAE pathogenesis (Chu et al., 2019; Plastini et al., 2020). M1-type cells primarily secrete pro-inflammatory factors, which exacerbate neuroinflammation and demyelinating injury, while M2-type cells promote remyelination and tissue repair by releasing anti-inflammatory factors. Thus, inhibiting M1 polarization or promoting M2 polarization may represent an important component in the prevention and treatment of MS. Recent research has identified several interventions that modulate this polarization process to exert therapeutic effects. For instance, baicalein alleviates EAE symptoms by inhibiting STAT1 activation, thereby blocking M1 polarization and reducing pro-inflammatory factor release (Ma et al., 2022). A ketogenic diet promotes the M1-to-M2 shift through regulation of the NF-κB/NLRP3 pathway and inhibition of HDAC3 and P2X7R, improving demyelinating pathology (Sun et al., 2023). Mdivi-1 induces M2 polarization by inhibiting Drp1 serine 616 phosphorylation and blocking the TLR2/4-GSK3β-NF-κB pathway, thereby reducing neuroinflammation (Liu et al., 2022b). Propranolol enhances phagocytic function and regulates cytokine balance by upregulating CX3CR1 and activating the Nrf2/HO-1 axis, thus inhibiting immune cell infiltration and Th17 differentiation (Pilipović et al., 2020). Additionally, BMSC-derived exosomes carrying miR-23b-3p reduce microglial pyroptosis by binding and inhibiting NEK7, thereby alleviating EAE severity (Wang et al., 2023). These studies demonstrate that regulating microglia/macrophage polarization is an effective approach to intervene in MS/EAE progression.

**Regulation of dendritic cell-mediated immune responses** As key antigen-presenting cells (APCs), dendritic cells play pivotal roles in initiating and regulating immune responses by presenting antigens to T cells, thereby mediating adaptive immunity. However, abnormal activation of dendritic cells can enhance T cell pathogenicity, promoting the development and progression of MS. Targeting dendritic cell maturation and antigen presentation can effectively inhibit abnormal T cell activation, thus reducing inflammatory responses in EAE. For example, Zhang et al. (2019) reported that scopoletin inhibits dendritic cell activation, downregulates co-stimulatory molecule expression, and reduces NF-κB phosphorylation. This leads to a significant reduction in the pathogenic responses of Th1 and Th17 cells and alleviates EAE severity. Similarly, Yang et al. (2020) found that astragaloside IV blocks CD4^+^ T cell differentiation into Th1/Th17 subsets by inhibiting dendritic cell maturation and antigen presentation, reducing pro-inflammatory factor release, thereby delaying EAE onset and alleviating neurological symptoms. These findings suggest that regulating dendritic cell function may offer potential strategies for MS immunological intervention.

**Regulation of B cell-mediated immune responses** B cells, or B lymphocytes, are essential for mediating humoral immune responses, playing central roles in immune defense through antigen recognition and antibody production (Li et al., 2018; Lazibat et al., 2018). However, in autoimmune diseases such as MS, B cells exhibit complex dual functions. As APCs, B cells can activate T cells and promote their proliferation through myelin antigen presentation, driving disease progression. Additionally, their secretion of autoantibodies, pro-inflammatory cytokines such as IL-6 and TNF-α, and formation of ectopic germinal centers in the CNS exacerbate neuroinflammation and tissue damage. On the other hand, B cells possess immunoregulatory capabilities, exerting protective effects by producing anti-inflammatory cytokines like IL-10 and IL-35 to suppress excessive inflammation. This functional duality makes B cells significant targets for MS research and therapy. Recent studies have identified several drugs and natural products that modulate B cell function in MS/EAE progression. For instance, Nuesslein-Hildesheim et al. (2023) reported that Bruton's tyrosine kinase inhibitor remibrutinib inhibits the autoreactivity of pathogenic B cells and exerts anti-inflammatory effects in microglia, thereby effectively delaying EAE progression. In contrast, Park et al. (2023) demonstrated that sphingosylphosphorylcholine reduces antibody production by inhibiting B cell differentiation into plasma cells and downregulating plasma cell-related gene expression. This highlights its potential value in regulating EAE immune responses and further uncovers the important role of B cells in EAE pathogenesis.

**Regulation of immune responses mediated by other immune cell subsets** Oligodendrocytes and astrocytes are essential glial cell populations in the CNS, playing critical roles in MS pathogenesis. Oligodendrocytes, originating from oligodendrocyte precursor cells (OPCs), are responsible for myelination and its maintenance. In MS, oligodendrocyte dysfunction leads to demyelination and subsequent neurological deficits. Astragalus polysaccharides protect neurons in EAE models by reducing neuroinflammation, inhibiting CD8^+^ T cell infiltration and IFN-γ production, and enhancing the differentiation of neural stem cells into oligodendrocytes (Zhao et al., 2024). Ginsenosides, on the other hand, promote OPC-mediated myelin repair and regeneration through modulation of the GSK3β/β-Catenin signaling pathway, thereby improving demyelinating pathology in EAE (Liu et al., 2022a). Astrocytes maintain CNS homeostasis under normal conditions by regulating synaptic function, ensuring BBB integrity, and supporting neural metabolism. In pathological states, however, they adopt a reactive phenotype, polarized into neurotoxic A1 astrocytes and neuroprotective A2 astrocytes, thus playing a dual role in MS pathology. Matrine derivatives mitigate EAE-induced neural damage by suppressing inflammatory astrocyte responses and A1 phenotype formation, while promoting synaptic stability and preserving BBB function (Fan et al., 2022). Isoliquiritigenin reduces the severity of EAE pathology by modulating the balance of reactive astrocyte phenotypes, inhibiting pro-inflammatory signals, and enhancing their protective capabilities (Zhang et al., 2024). Both oligodendrocytes and astrocytes are pivotal in MS pathogenesis: oligodendrocytes control myelin formation and repair, while astrocytes regulate BBB integrity and neuroinflammation. These findings highlight that compounds like Astragalus polysaccharides, ginsenosides, matrine derivatives, and isoliquiritigenin can alleviate EAE pathology by modulating glial cell functions, suggesting promising therapeutic targets and strategies for neurorepair in MS.

#### 3.2.2 Regulation of autophagy

Autophagy, a highly conserved degradation-recycling system within cells, plays a pivotal role in maintaining cellular homeostasis, stress responses, and developmental differentiation by eliminating dysfunctional organelles and macromolecules (Plaza-Zabala et al., 2017; Mizushima et al., 2008). Abnormal autophagy levels are closely associated with the pathogenesis and progression of various diseases, including neurological disorders.

In MS, autophagy exhibits complex, bidirectional regulatory effects. Moderate autophagy can reduce oxidative stress by eliminating reactive oxygen species and damaged organelles, while suppressing neuroinflammation through regulation of inflammatory pathways such as NF-κB and NLRP3. Simultaneously, it optimizes the cellular metabolic environment and promotes the expression of myelin components, including myelin basic protein, which enhances the survival and function of oligodendrocytes and astrocytes, thus synergistically supporting remyelination. However, under pathological conditions such as persistent endoplasmic reticulum stress, DNA damage, or excessive pro-inflammatory stimulation, autophagy can become dysregulated, exacerbating myelin structural damage and demyelination. This is achieved through disruption of immune cell homeostasis, excessive microglial activation, and abnormal Th17 cell differentiation.

Various drugs and natural products can modulate autophagy processes to intervene in MS/EAE progression. For example, fluoxetine and cannabidiol enhance autophagic flux by upregulating autophagy-related genes such as LC3II and Beclin-1, while inhibiting apoptosis markers like Bax/Bcl2, thereby reducing demyelinating damage in the cerebellar white matter and improving EAE scores (Akhavan Tavakoli et al., 2023). Neutrophil-derived nanovesicles (NDNVs) promote LC3-associated phagocytosis in microglia through upregulation of RUBCN via the NRF2 signaling pathway, facilitating myelin debris clearance and inflammation resolution, resulting in improved neurological function and preserved white matter integrity in EAE mice (Shen et al., 2022). Sildenafil regulates the AMPK-mTOR pathway through the eNOS-NO-cGMP axis, activating LC3-Beclin1-ATG5-mediated autophagy while exerting neuroprotective effects via the CREB-BDNF pathway, thus alleviating EAE symptoms (Duarte-Silva et al., 2021). Nicotinamide adenine dinucleotide activates autophagy by promoting LC3-II/I and Beclin1 expression and modulating Th1/Th17 cell differentiation, which reduces demyelination and axonal damage (Wang et al., 2021). Phloretin activates the Nrf2 pathway by promoting Keap1 degradation through AMPK-dependent autophagy activation, exerting antioxidant and anti-inflammatory effects (Dierckx et al., 2021).

#### 3.2.3 Regulation of gut microbiota and intestinal microecology

The gut microbiota, a highly diverse microbial community that colonizes the digestive tract, plays a critical role in maintaining health, with its imbalance linked to a variety of pathological conditions, including tumors, autoimmune diseases, metabolic disorders, and infections. Microbiota dysbiosis primarily mediates systemic pathological effects through three synergistic pathways: first, a reduction in beneficial bacteria compromises the intestinal epithelial barrier, facilitating the translocation of bacterial lipopolysaccharide, β-glucan, and bacterial DNA, which continuously activate pattern recognition receptors such as TLR4/NLRP3. This results in systemic low-grade inflammation marked by elevated TNF-α and IL-6, laying the inflammatory groundwork for conditions like metabolic syndrome and atherosclerosis (Shen et al., 2025). Second, altered microbial metabolic profiles induce abnormal polarization of dendritic cells and macrophages, disrupting the Th17/Treg balance and cytokine homeostasis between IL-17 and IL-10. This not only promotes autoimmune disease development but also exacerbates tumorigenesis and weakens mucosal immunity through STAT3 signaling pathways (Ivanov et al., 2008). Third, a reduction in microbial metabolites like short-chain fatty acids (e.g., butyrate) affects histone deacetylase activity, leading to imbalanced epithelial proliferation and apoptosis. Additionally, choline and carnitine metabolized by microbiota generate trimethylamine, which is converted to TMAO in the liver. TMAO activates NLRP3 inflammasomes in macrophages and endothelial cells, contributing to atherosclerosis (Shen et al., 2025). Abnormal secondary bile acid composition also interferes with FXR/TGR5 signaling, disrupting glucose and lipid metabolism, which is associated with the pathogenesis of non-alcoholic fatty liver disease and type 2 diabetes (Shen et al., 2025).

The gut microbiota also serves as an important antigen source, influencing the development of gut-associated lymphoid tissues and regulating immune cell functions, thereby having a profound impact on systemic immunity (Mueller et al., 2015). Studies have shown significant alterations in gut microbiota composition in patients with MS, highlighting the role of the microbiota-gut-brain axis in MS pathogenesis (Sommer and Bäckhed, 2013; Pröbstel and Baranzini, 2018; Ochoa-Repáraz et al., 2009). An abnormal microbiota structure may enhance peripheral Th17 immune responses and compromise BBB function through mechanisms such as intestinal barrier disruption, reduced production of butyrate and tryptophan metabolites, and increased release of pathogenic toxins, thereby exacerbating CNS demyelination and neurological damage.

Based on these mechanisms, several studies have explored microbiota-targeted intervention strategies in EAE models. For example, Liu et al. (2019) identified an abnormal gut microbiota structure in EAE mice and demonstrated that upregulating miR-30d could improve microbiota composition and alleviate disease severity. Al-Ghezi et al. (2019) reported that combined THC and CBD treatment reduced the abundance of the mucin-degrading bacterium Akkermansia muciniphila, decreased LPS synthesis, and promoted short-chain fatty acid production, such as butyrate. This regulatory effect on immune balance led to reduced levels of IL-17 and IFN-γ, increased anti-inflammatory factors IL-10 and TGF-β, and ultimately alleviated EAE clinical symptoms. Prado et al. (2023) found that selective GPR43 agonists could inhibit the pro-inflammatory phenotype of colonic TCRαβ^+^ T cells and block their migration to the CNS via CXCR3, significantly delaying EAE progression. Additionally, natural products such as oxymatrine (Zhang et al., 2023), matrine (Dou et al., 2024), and naringenin (Liu et al., 2023) have shown potential in improving EAE symptoms by regulating gut microbiota structure.

#### 3.2.4 Regulation of blood-brain barrier integrity

The BBB plays a critical role in maintaining CNS homeostasis, with its integrity relying on tight junction proteins (such as occludin, claudin-5, ZO-1, and ZO-2) and adhesion molecules that establish stringent connections between brain microvascular endothelial cells. Disruption of the BBB is a key feature in the pathogenesis of MS (Chen and Shifman, 2019), where activated T lymphocytes, B lymphocytes, and macrophages release inflammatory cytokines and mediators that damage tight junctions, leading to increased barrier permeability and CNS dysfunction.

Recent studies have highlighted several naturally active components with protective effects on the BBB. Oh et al. (2024) demonstrated that ginsenoside Rg3 can prevent abnormal alterations in key BBB components in EAE models and under LPS stimulation. These alterations include astrocyte activation, upregulation of cell adhesion molecules like platelet endothelial cell adhesion molecule-1, and degradation of tight junction proteins. Ginsenoside Rg3 also alleviates demyelinating damage by inhibiting the TLR4/MyD88/NF-κB signaling pathway. Niu et al. (2021) found that naringenin reduces dendritic cell chemotaxis by downregulating chemokine CCL19 and its receptor CCR7 expression, thereby decreasing pathogenic T cell migration to the CNS and mitigating their harmful effects on tight junction proteins ZO-1 and occludin, thus preserving BBB integrity. Furthermore, Lee et al. (2021) showed that Korean red ginseng extract, rich in ginsenoside Rg3, protects the BBB by regulating NADPH oxidase 2 and 4 expression, leading to improved behavioral and neuropathological outcomes in EAE models.

#### 3.2.5 Regulation of NLRP3 inflammasome activation and pyroptosis

Inflammasomes are critical multi-protein complexes in innate immune responses (Barnett et al., 2023; Desu et al., 2020), composed of sensor proteins, the adaptor protein ASC, and the effector protein pro-caspase-1 (Broz and Dixit, 2016). These complexes play vital roles in immune surveillance. Depending on the type of sensor protein, inflammasomes can be categorized into NLRP3, AIM2, NLRC4, and Pyrin types. Among these, the abnormal activation of the NLRP3 inflammasome has been identified as a central pathogenic factor in MS and its animal model, EAE (Gris et al., 2010; Olcum et al., 2020).

In MS/EAE pathogenesis, excessive activation of the NLRP3 inflammasome promotes the maturation of IL-1β and IL-18 and triggers Gasdermin-D-mediated pyroptosis, compromising the BBB and continuously recruiting Th17 cells to the CNS. Additionally, ASC specks can be transferred between cells, transforming localized inflammation into widespread demyelinating lesions, thereby accelerating disease progression.

Recent studies have demonstrated that several synthetic drugs and natural compounds exert therapeutic effects in EAE by regulating NLRP3 inflammasome activation and pyroptosis. For instance, Li et al. (2022) reported that Manoalide inhibits inflammasome assembly by covalently binding to lysine residues on the NLRP3 protein, blocking the interaction between NEK7 and NLRP3, improving ion homeostasis and mitochondrial function, and significantly delaying EAE progression. Yu et al. (2020) showed that Bixin alleviates neuroinflammation and demyelination by inhibiting oxidative stress and the TXNIP/NLRP3 pathway, activating NRF2 signaling, and modulating the Th1/Th17 balance. Huang et al. (2023) found that tivantinib specifically blocks NLRP3 assembly and activation by reducing its ATPase activity, independent of c-Met inhibition, thus alleviating EAE pathological progression. Motawi et al. (2023) confirmed that CLM inhibits oxidative stress and NLRP3 signaling through the Nrf2/HO-1 pathway, reducing cytokine release and pyroptosis, and improving EAE behavioral scores and neurological symptoms. Additionally, Song et al. (2022) reported that liraglutide, a GLP-1 receptor agonist, significantly alleviates CNS inflammation and demyelinating injury in EAE by inhibiting the NLRP3 pathway and restoring autophagy at doses that do not induce hypoglycemia.

#### 3.2.6 Mechanistic crosstalk and synergistic regulation

**Figure 3:**
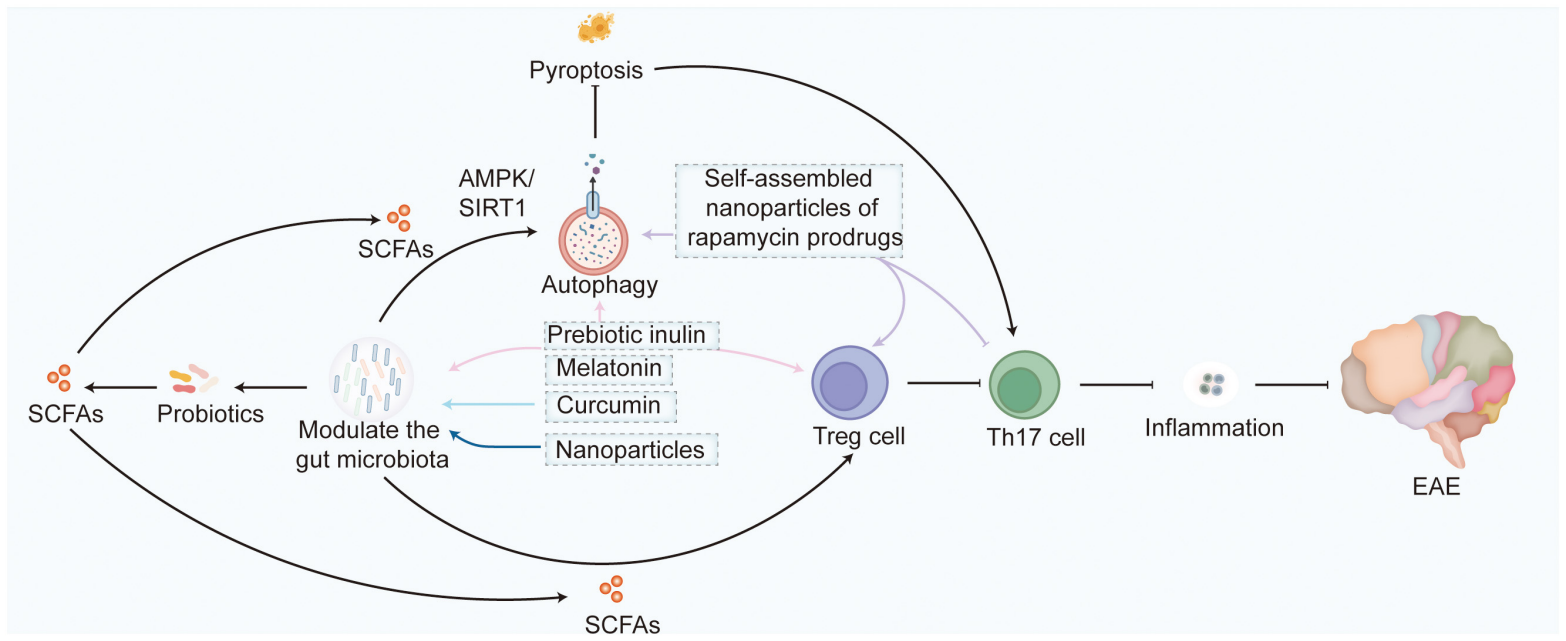
Certain compounds exhibit multi-mechanism synergistic effects during multiple sclerosis (MS)/experimental autoimmune encephalomyelitis (EAE) intervention, achieving more comprehensive regulation of disease processes by modulating multiple pathways, including immunity, metabolism, and autophagy. Additional abbreviations: Th17, T helper type 17; Treg, regulatory T cell; AMPK, AMP-activated protein kinase; SIRT1, sirtuin 1; SCFAs, short-chain fatty acids; EAE, experimental autoimmune encephalomyelitis.

Systematic studies have shown that certain compounds exhibit multi-mechanism synergistic effects during MS/EAE intervention, achieving more comprehensive regulation of disease processes by modulating multiple pathways, including immunity, metabolism, and autophagy (Figure 3). Li et al. (2024) found that prebiotic inulin regulates gut microbiota structure, promotes short-chain fatty acids (SCFAs) production, and enhances Treg cell function. It also activates autophagy, clears damaged organelles, and inhibits pyroptosis, ultimately reducing neuroinflammation and neuronal damage by inhibiting pro-inflammatory T cell activation. Sadek et al. (2024) reported that curcumin optimizes microbiota composition and increases beneficial SCFAs, enhancing Treg function. It also activates autophagy and reduces pyroptosis through the AMPK/SIRT1 pathway, effectively inhibiting Th17-mediated CNS inflammation. Zhang et al. (2025) developed rapamycin prodrug self-assembling nanoparticles that activate autophagy and clear damaged organelles through mTOR inhibition while enhancing Treg activity, inhibiting Th17 differentiation, and suppressing inflammation through gut microbiota regulation, demonstrating multi-pathway synergistic therapeutic effects. Álvarez-López et al. (2024) showed that melatonin significantly inhibits Th17 activation and reduces neuroinflammatory damage in combination with methylprednisolone by regulating gut microecology, promoting Treg function, and activating autophagy, thereby slowing MS progression.

## 4 Summary and perspectives

MS, a leading cause of non-traumatic disability in young adults, significantly impairs quality of life due to recurrent relapses and progressive neurological dysfunction. Although its etiology remains incompletely understood, currently approved therapies–largely centered on immunomodulation–primarily aim to reduce inflammatory activity and relapse frequency, yet remain limited in halting long-term progression or promoting effective neurorepair. If emerging mechanisms are to be considered as starting points for novel therapeutic strategies, their potential clinical applicability and translational feasibility warrant explicit discussion.

Recent advances in elucidating MS pathogenesis have identified multiple interconnected processes–including immune dysregulation, autophagy imbalance, gut-brain axis disturbances, blood-brain barrier (BBB) disruption, and NLRP3 inflammasome-mediated pyroptosis–as potential mechanistic entry points for therapeutic innovation. In this context, bioactive compounds derived from traditional medicines have attracted attention because of their capacity to modulate multiple targets simultaneously. This review systematically summarizes preclinical findings based on the EAE model, focusing on how these mechanisms may serve not merely as descriptive pathways but as conceptual frameworks for future drug development. For each pathway, we discuss its potential as a therapeutic initiation point–for example, selective immune rebalancing rather than broad immunosuppression, restoration of autophagic homeostasis to support neuroprotection, modulation of gut microecology to influence systemic and CNS inflammation, preservation of BBB integrity to limit immune cell infiltration, and inhibition of NLRP3-driven pyroptosis to attenuate neuroinflammatory amplification.

While certain clinically used agents, such as dimethyl fumarate, have demonstrated immunomodulatory effects that partially intersect with these mechanisms, several emerging targets–particularly autophagy regulation and microbiota-brain axis modulation–have not yet yielded clinically validated drugs, underscoring both opportunity and uncertainty. Importantly, EAE remains a valuable experimental platform for mechanistic exploration and hypothesis generation, but it does not fully replicate the metabolic complexity, heterogeneity, or long-term progression of human MS. Numerous compounds effective in EAE have failed in subsequent clinical trials. Therefore, the evidence discussed herein should be interpreted as mechanistic and exploratory rather than confirmatory of clinical efficacy.

Future MS therapeutic research should move beyond descriptive mechanistic associations and critically evaluate how each pathway can be translated into rational, mechanism-based intervention strategies. Emphasis should be placed on multi-target drug design grounded in defined biological networks, rigorous translational validation from animal models to human studies, and refinement of precision medicine approaches. Strengthening the translational continuum from experimental findings to clinical application will be essential to meaningfully advance MS disease management and ultimately improve patient outcomes.
